# Self-reported Disability Among Recently Resettled Refugees in the United States: Results from the National Annual Survey of Refugees

**DOI:** 10.1007/s10903-023-01580-4

**Published:** 2023-12-18

**Authors:** Mehak Kaur, Lily Kamalyan, Dania Abubaker, Rawan Alheresh, Tala Al-Rousan

**Affiliations:** 1 Department of Population and Public Health Sciences, Keck School of Medicine, University of Southern California, Los Angeles, CA USA; 2 Department of Psychiatry, HIV Neurobehavioral Research Program, University of California, San Diego, CA USA; 3 San Diego Joint Doctoral Program in Clinical Psychology, San Diego State University, University of California, San Diego, CA USA; 4 Herbert Wertheim School of Public Health, University of California San Diego, La Jolla, CA USA; 5 MGH Institute of Health Professions, Boston, MA USA

**Keywords:** Refugee, Disability, Healthcare Access, Mental Health, Annual Survey of Refugees (ASR)

## Abstract

The prevalence rates and correlates of mental or physical disability among recently resettled refugees, who undergo strenuous journeys before arriving in the US, remain unknown, masking potential health disparities. Self-reported disability was measured by the 2018 Annual Survey of Refugees (ASR), and defined as having a physical, mental, or other health condition for more than 6 months that precluded one from working. Prevalence rates of self-reported disability and sample correlates were investigated using descriptive and logistic regression analyses. Of N = 4259 participating refugees in ASR (Mean Age = 28.2, SD = 17.2; 52.5% male), 2875 responded to the disability question and 21.4% reported disability. About 33.7% were born in the Middle East region, 29.5% had no formal education, and 35% had an income of less than $15,000. Age (OR = 1.06, 95% Confidence Interval (CI) [1.06,1.07], p < 0.001), region of birth (OR = 1.82, 95% CI [1.31, 2.51], p < 0.001), employment status (OR = 3.31, 95% CI [2.67, 4.11], p < 0.001), and receiving food stamps (OR = 2.09, 95% CI [1.66, 2.62], p < 0.001) were associated with self-reported disability. Disability levels among refugees recently resettled in the United States are comparable to national disability rates in the US. Our results suggest that multiple aspects of the refugee experience (i.e., demographics, socioeconomic status, contextual migration history) need to be considered to understand the risk for health outcomes. Future investigations of disabilities in diverse refugee populations over time and tailored public health interventions to mitigate potential risk factors are warranted to promote health equity.

## Background

Ever since the Refugee Act of 1980, the United States (US) has become home to approximately 3.1 million refugees [1]. In 2021, the US was the world’s largest recipient of new individual refugee applications [2]. Due to the ongoing migration crisis following the withdrawal of US from Afghanistan and the war in Ukraine, this trend is expected to grow. Today, 1 in 78 people are forcibly displaced [3] and of those, about 15% (12 million) are expected to have one or more disabilities [4].

The United Nations Convention on the Rights of Persons with Disabilities defines a disability as having a chronic physical, mental, intellectual, or sensory impairment that, when combined with other obstacles, may prevent an individual from fully and equally participating in society [5]. It is estimated that about 15% of the world’s population has some form of disability, with about 61 million US adults alone [6, 7]. By the end of 2021, there were approximately 1.2 million additional individuals with disabilities in the US due to the COVID-19 pandemic [8]. These rates of disability are related to increased expenses on the US healthcare budget, with more than 36% of the entire budget (about $868 billion) spent on disability services each year [9]. Despite the increasing number of refugees and the exponential increase in the disability burden in the US in recent years, there is limited literature on the prevalence of disability among refugees who have resettled in the US.

Due to the unique set of circumstances surrounding refugees’ escape from their home countries and resettlement in their new host countries, they face several challenges that increase their risk of developing mental or physical disabilities at various points in their lives, including physical and psychological trauma and fragmented access to healthcare, education, and social services. However, refugees are frequently overlooked as a distinct population suffering from health disparities [10–12]. Refugees are also more likely to take on risky jobs like working in assembly line/packaging factories, working in harsh conditions without proper equipment or be frontline workers such as registered nurses or janitors which was evident during the pandemic as well [13, 14]. Tools like the Global Activity Limitation Indicator (GALI) and disability metrics in the American Community Survey (ACS) are used to measure disability through national surveys where participants are asked whether a chronic health condition limits their participation in everyday activities, but these tools employ questions aimed at the general population and do not take into account the specific experiences of refugees [15, 16]. The Annual Survey of Refugees (ASR) is a unique government-sponsored national survey administered to recently resettled refugees through the US Refugee Admission Program [17]. The ASR aims to better understand the experiences of refugees during the first five years after resettlement in the US [17]. In this study, we used the 2018 ASR dataset to examine potential factors associated with self-reported disability among recently resettled refugees.

## Methods

### Participants and Data Collection

Secondary analysis was performed using the data collected from the 2018 Annual Survey of Refugees (ASR), conducted by the Office of Refugee Resettlement in early 2019 [17, 18]. This survey included refugees that entered the US during the federal fiscal years (i.e., October through September) of 2013–2017 and emphasized their first five years of resettlement. Participants were required to be at least 16 years old at the time of the interview to participate in the survey. The ASR survey used a stratified probability design to select households and people within households to participate. Stratification factors included age at arrival, gender, year of arrival, geographic region of resettlement, and household size at the time of arrival. Data were collected from 4259 eligible refugees, of which 52.5% were male and 47.5% were female. The survey was translated into sixteen non-English languages which covered 73% of the 2013–2017 survey population [17]. Since the study did not meet the criteria for human subjects research, the Institutional Review Board at the author’s institution determined that no ethical approval was required.

### Measures

The outcome variable of self-reported disability was collected using the following survey question: “Does the refugee have a physical, mental, or other health condition that has lasted for 6 or more months and which either limits the kind or amount of work the refugee can do at a job or it prevents the refugee from working at a job?” The response options included *no, yes, do not know*, and *refused*. Responses from both limited work and no work were recorded separately as two variables in the original dataset but were combined in this analysis into one binary variable to determine overall physical and mental disability. “Yes” responses to either or both questions were recorded as a yes, and the “no” responses to both questions were recorded as a no. Individuals who answered “do not know” or those who refused to answer were excluded from the analysis along with the missing values (n = 1384), leaving a total sample of 2875.

The potential factors of self-reported disability were chosen from available data based on previous literature: age, gender, marital status, permanent resident status, year of arrival, health coverage, employment status, country of birth, resettlement region, English proficiency, source of medical care, annual income, level of education, and food stamps. Marital status was recoded into two categories (i.e., currently married and not currently married) from nine original categories. Insurance coverage was condensed into three categories (i.e., covered in all months, not covered for a certain number of months, and not covered at all). The region of birth was derived from the country of birth, and countries were recoded based on their geographical region (i.e., Asia, Africa, Middle East, and Other). The highest level of education obtained by refugees before coming to the US was also recoded into categories of none, primary, secondary, higher (university and medical degrees), and other (training/technical school) education. Finally, annual income was converted into a categorical variable with 4 levels for ease of interpretation.

### Analysis

All analyses were weighted using the ASR person-level analytic weights. For each of the factor variables, the frequency and percentage of disability were determined. First, Chi-square tests were conducted to examine the association between self-reported disability status and each potential factor. Simple logistic regressions were performed to obtain crude odds ratios for each factor. Second, a multiple logistic regression model was developed that included all fourteen factors. Multicollinearity analyses were run, and variance inflation factor (VIF) values were less than 5. Each factor’s effect on the multivariable model was evaluated using a manual stepwise method. Any variables that were not significant at *p* < 0.05 were removed, and the model was re-run. In addition, to determine overall model fitness, we used the Bayesian information criterion (BIC) value. The following logistic regression assumptions were met: each observation was independent; multicollinearity was not present in the final model and the linearity assumption was met by the age variable. The Statistical Package for Social Sciences (SPSS), version 28.0, was used with a significance level of α = 0.05 to perform the statistical analysis.

## Results

### Descriptive Analysis (Table 1)

Descriptive statistics for the sample are reported in Table 1 by disability status. Out of the total 2875 responses, 21.36% (n = 614) reported having a condition that limited or prevented them from working, whereas 78.64% (n = 2261) denied having a disability.

**Table 1:** Socio-demographic characteristics of the study population by self-reported disability

Socio-demographic Characteristics	Total *n (%)* ^*^ 2875	Disability *n (%)* ^*^ 614 (21.4)	No Disability *n (%)* ^*^ 2261 (78.6)	*p-value*
**Age, mean (SD)**		46.81 (16.1)	33.25 (12.4)	< 0.001
**Gender**				< 0.001
Male	1532 (53.3)	289 (47.1)	1243 (55.0)	
Female	1342 (46.7)	325 (52.9)	1017 (45.0)	
**Marital Status**				< 0.001
Currently Married	1615 (56.4)	397 (64.8)	1218 (54.1)	
Not currently married	1251 (43.6)	216 (35.2)	1035 (45.9)	
**Highest Level of Education (before coming to the US)**				< 0.001
None	831 (29.5)	228 (37.9)	603 (27.2)	
Primary	698 (24.7)	145 (24.1)	553 (24.9)	
Secondary	771 (27.3)	125 (20.8)	646 (29.1)	
Higher	292 (10.4)	52 (8.7)	240 (10.8)	
Other	229 (8.1)	51 (8.5)	178 (8.0)	
**Annual Income**				< 0.001
Less than $15,000	348 (35.0)	75 (54.0)	273 (31.9)	
$15,000 to $24,999	233 (23.4)	34 (24.5)	199 (23.2)	
$25,000 to $34,999	199 (20.0)	16 (11.5)	183 (21.4)	
More than $35,000	215 (21.6)	14 (10.1)	201 (23.5)	
**Region of Birth**				< 0.001
Asia	722 (25.2)	156 (25.6)	566 (25.1)	
Africa	710 (24.8)	110 (18.1)	600 (26.6)	
Middle East	967 (33.7)	256 (42.0)	711 (31.5)	
Other	469 (16.4)	87 (14.3)	382 (16.9)	
**Year of Arrival**				0.813
2013 or earlier	730 (25.9)	147 (24.9)	583 (26.2)	
2014	601 (21.3)	128 (21.7)	473 (21.2)	
2015	470 (16.7)	99 (16.8)	371 (16.7)	
2016	748 (26.6)	153 (25.9)	595 (26.7)	
2017 or later	267 (9.5)	63 (10.7)	204 (9.2)	
**Resettlement Region (US)**				< 0.001
Northeast	446 (15.7)	121 (20.2)	325 (14.5)	
South	882 (31.1)	144 (24.1)	738 (33.0)	
Midwest	766 (27.0)	168 (28.1)	598 (26.8)	
West	738 (26.1)	165 (27.6)	573 (25.6)	
**Current English Proficiency**				< 0.001
Yes	1495 (52.2)	170 (27.8)	1325 (58.8)	
No	1370 (47.8)	441 (72.2)	929 (41.2)	
**Source of Medical Care**				< 0.001
No regular source	522 (18.7)	65 (10.8)	457 (20.8)	
Private physician	739 (26.4)	163 (27.1)	576 (26.3)	
Emergency Room	402 (14.4)	105 (17.5)	297 (13.5)	
Health Clinic	834 (29.8)	168 (28.0)	666 (30.4)	
Folk Healer	163 (5.8)	58 (9.7)	105 (4.8)	
Other	134 (4.8)	42 (7.0)	92 (4.2)	
**Insurance Coverage**				< 0.001
Covered in all months	1568 (58.6)	402 (69.2)	1166 (55.6)	
Not covered for a certain number of months	286 (10.7)	70 (12.0)	216 (10.3)	
Not covered at all	823 (30.7)	109 (18.8)	714 (34.1)	
**Worked Last Week**				< 0.001
Yes	1618 (56.4)	178 (29.0)	1440 (63.8)	
No	1252 (43.6)	435 (71.0)	817 (36.2)	
**Applied to be a Permanent US Resident**				0.290
Yes	2402 (84.2)	521 (85.6)	1881 (83.8)	
No	452 (15.8)	88 (14.4)	364 (16.2)	
**Received Food Stamps**				< 0.001
Yes	1558 (54.8)	446 (73.0)	1112 (49.8)	
No	1284 (45.2)	165 (27.0)	1119 (50.2)	

*Note*: *n (number of participants) and *%* are weighted according to the ASR person−level analytic weights

All sample characteristics were found to be significantly related to disability status except for the year of arrival and permanent residence status (Table 1). More females than males reported having a disability (52.9% vs. 47.1%) and the mean age of refugees that reported having a disability was 46.81 years old (*SD* = 16.10). Refugees born in the Middle East had a much higher frequency of self-reported disability (42%) as compared to the refugees born in other parts of the world (Asia = 25.6%, Africa = 18.1%, Other = 14.3%). Refugees who resettled in the Midwest had the highest prevalence of self-reported disability (28.1%) as compared to the Northeast (20.2%), South (24.1%) and West (27.6%). Refugees who were not proficient in English had a higher percentage of self-reported disability (72%) as compared to those who were English proficient (27.8%).

Refugees who used health clinics as a source of medical care had the greatest prevalence of disability (28.0%) as compared to the other sources including private physicians and emergency rooms. Refugees that had insurance coverage all year had a greater prevalence of self-reported disability (69.2%) than other levels of health care coverage. Married refugees had a higher prevalence of disability (64.8%) as compared to those who were not (35.2%). Refugees who were unemployed (71.0%) and received food stamps (73.0%) had a high frequency of self-reported disability. Refugees who received no education before coming to the US had a much higher prevalence of disability (37.9%) than refugees who received any education. Refugees with an annual income of $15,000 or less had a much higher prevalence of self-reported disability (54.0%) as compared to the other income groups.

### Self-Reported Disability by Arrival Year and Country of Birth (Fig. 1)

**Fig. 1:**
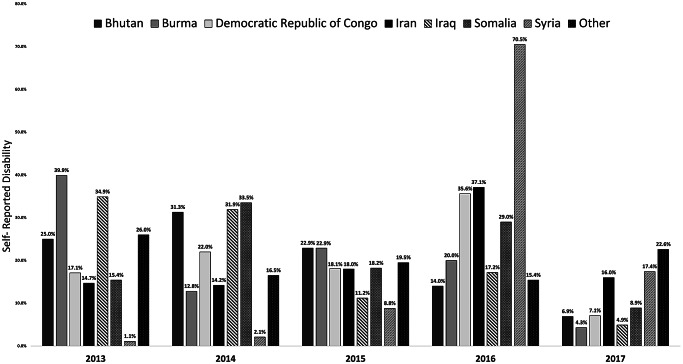
Self-Reported disability by arrival year and country of birth

Figure 1 depicts the frequency of self-reported disability by arrival year and country of birth. Across the five years that refugees entered the US, the percentage of self-reported disability has been declining for those born in Bhutan (2014 = 31%, 2017 = 6.9%), Burma (2013 = 39.9%, 2017 = 4.3%), and Iraq (2013 = 34.9%, 2017 = 4.9%). For those born in the Democratic Republic of Congo, the prevalence of self-reported disability increased from 2013 (17.1%) to peak in 2016 (35.6%), before a steep reduction in 2017 (7.1%). Those born in Syria reported less than 10% of disability from 2013 to 2015, however, those from Syria who arrived in 2016 had the highest prevalence of disability across all countries and arrival cohorts (70.5%). Those who were born in ‘Other’ countries reported the highest level of disability in 2013 (26%).

### Crude Logistic Regression (Table 2)

Out of all the potential factors, year of arrival and permanent residence status were not statistically significant (*p* > 0.05) (Table 2). With every ten years increase in age, the odds of self-reported disability increased by 7% (95% Confidence Interval (CI) [1.06, 1.07], *p* < 0.001). As compared to males, females had greater odds of self-reported disability (OR = 1.37, 95% CI [1.15, 1.64], *p* < 0.001). Refugees born in the Middle East had 57% higher odds of disability as compared to those born in ‘Other’ regions (95% CI [1.19, 2.06], *p* = 0.001). Refugees who resettled in the Midwest had 24% lower odds of disability than refugees who resettled in the Northeast (95% CI [0.58,0.99], *p* = 0.04) whereas refugees who resettled in the South had 47% lower odds of disability (95% CI [0.40, 0.69], *p* < 0.001). Refugees who were not proficient in English had 269% greater odds of disability than those who were English proficient (95% CI [3.03, 4.49], *p* < 0.001).

**Table 2:** Associations between socio-demographic factors and self-reported disability

Socio-demographic Factors	Disability vs. No Disability Odds Ratio (95% Confidence Interval)	
	Crude	Adjusted
**Age**	1.07 (1.06, 1.07)*	1.06 (1.06, 1.07)*
**Gender**		
Female	1.37 (1.15, 1.64)*	-
Male	1 (ref)	-
**Marital Status**		
Currently married	1 (ref)	-
Not currently married	0.64 (0.53, 0.77)*	-
**Highest Level of Education (before coming to the US)**		
None	1.44 (1.14, 1.83)*	-
Primary	1 (ref)	-
Secondary	0.74 (0.57, 0.96)*	-
Higher	0.82 (0.58, 1.17)	-
Other	1.08 (0.75, 1.55)	-
**Annual Income before taxes**		
Less than $15,000	4.04 (2.20, 7.40)*	-
$15,000 to $24,999	2.56 (1.32, 4.93)*	-
$25,000 to $34,999	1.33 (0.63, 2.79)	-
More than $35,000	1 (ref)	-
**Region of Birth**		
Asia	1.20 (0.90, 1.61)	1.34 (0.95, 1.91)
Africa	0.80 (0.59, 1.09)	1.39 (0.97, 1.99)
Middle East	1.57 (1.19, 2.06)*	1.82 (1.31, 2.51)*
Other	1 (ref)	-
**Year of Arrival**		
2013 or earlier	1 (ref)	-
2014	1.07 (0.82, 1.40)	-
2015	1.06 (0.80, 1.42)	-
2016	1.03 (0.80, 1.32)	-
2017 or later	1.23 (0.88, 1.72)	-
**Resettlement Region (US)**		
Northeast	1 (ref)	-
South	0.53 (0.40, 0.69)*	-
Midwest	0.76 (0.58, 0.99)*	-
West	0.77 (0.59, 1.02)	-
**Current English Proficiency**		
Yes	1 (ref)	-
No	3.69 (3.03, 4.49)*	-
**Source of Medical Care**		
No regular source	1 (ref)	-
Private physician	1.97 (1.44, 2.69)*	-
Emergency Room	2.48 (1.76, 3.49)*	-
Health Clinic	1.77 (1.30, 2.40)*	-
Folk Healer	3.87 (2.57, 5.85)*	-
Other	3.17 (2.02, 4.97)*	-
**Insurance Coverage**		
Covered all year	1 (ref)	-
Not covered for a certain number of months	0.94 (0.70, 1.26)	-
Not covered at all	0.44 (0.35, 0.56)*	-
**Worked Last Week**		
Yes	1 (ref)	-
No	4.30 (3.54, 5.22)*	3.31 (2.67, 4.11)*
**Applied to be a Permanent US Resident**		
Yes	1.14 (0.89, 1.47)	-
No	1 (ref)	-
**Received Food Stamps**		
Yes	2.72 (2.23, 3.31)*	2.09 (1.66, 2.62)*
No	1 (ref)	-

Note: * depicts statistical significance (p<0.05)

Refugees without any insurance coverage had 56% lower odds of disability as compared to refugees who are covered all year (95% CI [0.35, 0.56], *p* < 0.001). As compared to married refugees, those who were not married had lower odds of disability (OR = 0.64 [0.53, 0.77], *p* < 0.001). Unemployed refugees had greater odds of disability as compared to those who were employed (OR = 4.30 [3.54, 5.22], *p* < 0.001). Refugees who received food stamps had 172% greater odds of disability (95% CI [2.23, 3.31], *p* < 0.001). The odds of self-reported disability decreased with increasing annual income. Refugees with an annual income of $15,000 or less had higher odds of disability as compared to refugees who made more than $35,000 a year (OR = 4.04 [2.20, 7.40], *p* < 0.001). All the various sources of medical care had lower odds of disability as compared to having no source of medical care. Refugees who reported folk healers as their source of medical care had 287% greater odds of disability as compared to those who did not have a regular source of medical care (95% CI [2.57, 5.85], *p* < 0.001). As compared to refugees who had primary education, those with no education had higher odds of reporting disability (OR = 1.44 [1.14, 1.83], *p* < 0.001) whereas those with secondary education had lower odds of reporting disability (OR = 0.74 [0.57, 0.96], *p* = 0.03).

### Multivariable Logistic Regression (Table 2)

The results from the multivariable logistic regression models for self-reported disability are provided in Table 2. Despite the non-significance of the year of arrival and permanent residence status at the crude odds level, they were added to the final model due to their clinical significance. For our final model (BIC = 1487.16), age, region of birth, employment status, and food stamps were all significantly and independently related to self-reported disability. For every ten-year increase in age, the odds of self-reported disability increased by 6% (95% CI [1.06, 1.07], *p* < 0.001). Refugees born in the Middle East had 87% higher odds of reporting disability as compared to the ones born in the ‘Other’ region (95% CI [1.31, 2.51], *p* < 0.001). Unemployed refugees had 231% greater odds of disability as compared to employed refugees (95% CI [2.67, 4.11], *p* < 0.001). Refugees who received food stamps had 109% higher odds of reporting disability as compared to the ones who did not receive them (95% CI [1.66, 2.62], *p* < 0.001).

## Discussion

To our knowledge, this is the first paper to examine factors associated with self-reported disability that limits or prevents someone from working at a job using a nationally representative sample of refugees recently resettled in the US. Our results revealed that disability rates among refugees varied based on their country of origin and year of arrival in the US, reflecting the global events of the time. For example, the refugees from Syria who arrived in the US in 2016 had the highest prevalence of disability as compared to others which can be explained by the fact that 2012 to 2015 were the most violent years of the Syrian war [19, 20]. We also found that multiple demographic, socioeconomic, and migration factors were univariably associated with higher rates of self-reported disability. In multivariable analyses, being older, born in the Middle East, recently unemployed, and receiving food stamps were independently and significantly associated with higher rates of self-reported disability. Our results focus on unmasking the health disparities that arise post-resettlement and highlight the various challenges that refugees face in the new environment.

Our results showed that being born in the Middle East region was associated with disability in the recently resettled refugees of this sample. This is unsurprising given the contextual nature of the wars in the Middle East between 2011 and 2016, coinciding with the time of the ASR survey. The wars in Iraq and Syria have contributed to mass displacement from the Middle East and were some of the most brutal armed conflicts in recent history [21, 22]. Previous studies have shown that compared to refugees from other regions, refugees from the Middle East region face multiple challenges that place them at higher risk of developing various physical and mental health issues [23, 24]. One of the major challenges specific to Middle Eastern refugees is their journey to the host country, which is not linear. Many refugees from the Middle East undertake dangerous journeys such as walking at night in the desert to cross to other countries such as Jordan or riding busses at night to forested areas and then taking overcrowded boats to cross the sea to Greece [25]. Many refugees including the ones from the Middle East may have to stay in multiple refugee camps, which is noteworthy, as refugee camps have poor living conditions which are not only associated with the development of physical and mental health issues but also perpetuate existing ones [2, 26]. Our results also indicated that age was statistically significantly associated with disability among this population, which is not surprising as aging and its associated general decline in health status is often associated with a higher chance of developing a disability [27]. Therefore, having programs specific to older refugees that can improve their quality of life are crucial.

Refugees who were unemployed also reported higher rates of disability. A recent meta-analysis of longitudinal studies on the relationship between mental well-being and unemployment found that customized interventions for promoting employment in individuals with a disability are the most effective [28]. Further research is needed to understand the potential connection between disability and employment among this population with very limited occupational options. Finally, food stamps were also found to be significantly associated with greater disability prevalence among this sample of refugees. Refugees that have resettled in the US reportedly have higher food insecurity as compared to the national average [29]. Thus, our results indicate that refugees of lower socioeconomic status who require assistance from supplemental programs are more likely to report a disability, which is consistent with previous literature [24]. However, this relationship may also be related to employment status, as low income may be a result of unemployment, and both low income and unemployment are reasons for individuals to sign up for the food stamp program [30]. There are also socioeconomic barriers to accessing health resources that need to be accounted for among recently resettled refugees since the majority of them have difficulty establishing financial security. Future longitudinal research can help further our understanding of barriers to disability justice among refugees over the life course.

Our study is the first to report on disability levels from a nationally representative sample of recent arrivals of refugees in the US, but notable limitations are worth mentioning. First, considering that this was cross-sectional data, we can only imply correlation and not causation from our results. Second, the ASR has a high non-response rate of 79%, which means that future surveys should consider recruitment strategies that may ensure a larger, more representative sample. Third, there is only one question in the survey that asks about having a physical and or mental health problem, limiting the various types and severity of disability among refugees which may not be an accurate measure of disability given the various cultural and language backgrounds of refugees and how they perceive this question. However, the Global Activity Limitation Indicator (GALI) has been validated in the literature as a one-item measure for disability [15]. Thus, we believe that our measure is still a valid overall measure of disability. Fourth, of the 4259 eligible refugees, about 32.5% did not or chose not to respond to the disability question. This means that about one-third of the sample was excluded from the analysis which could have potentially skewed our results. Fifth, despite knowing the time of arrival of refugees, the survey does not clarify whether the refugee developed the disability before or after coming to the US, which is a crucial piece of information. Finally, another limitation is the lack of information about the health conditions of the refugees, such as chronic health conditions, medications, treatments, and the number of visits to the doctor. Further research among this community should gather data on individual health factors that may be a result of their refugee experience, which would allow for a more detailed analysis of mechanisms of physical or mental disability, thus leading to more tailored care and health policies for this population.

## Data Availability

The original data source can be obtained at the following DOI: 10.3886/E131025V1.
